# *Prosopis africana* protects against heavy metal mixture-induced pulmonary and hematological toxicity via nuclear factor erythroid 2-related factor 2/ nuclear factor-kappa B/ caspase-3 signaling

**DOI:** 10.5114/bta/223960

**Published:** 2026-09-09

**Authors:** Harrison A. Ozoani, Kenneth O. Okolo, Baridoo Donatus Dooka, Cecilia N. Obasi, Prosper Manu Abdulai, Orish E. Orisakwe

**Affiliations:** 1 African Centre of Excellence for Public Health and Toxicological Research, University of Port Harcourt, Choba, Nigeria; 2 Department of Pharmacology and Toxicology, Faculty of Pharmacy, Enugu State University of Science and Technology, Enugu, Nigeria; 3 Department of Experimental Pharmacology and Toxicology, Faculty of Pharmaceutical Sciences, University of Port Harcourt, Port Harcourt, Nigeria; 4 Advanced Research Centre, European University of Lefke, Lefke, Northern Cyprus

**Keywords:** *Prosopis africana*, heavy metal toxicity, Nrf2/ NF-κB/ caspase-3 signaling, oxidative stress, hematotoxicity, metal bioaccumulation

## Abstract

**Background:**

Heavy metal mixture (HMM) exposure poses severe health risks by inducing oxidative stress and inflammation. The limitations of conventional chelation therapy necessitate exploring natural alternatives. Although *Prosopis africana* (PA) has antioxidant properties, its potential as a protective agent remains underexplored. This study investigated the protective mechanisms of PA against lead/ cadmium/arsenic-induced pulmonary and hematological toxicity.

**Materials and methods:**

Rats were treated with HMM alone or combined with PA (500, 1000, or 1500 mg/kg). The evaluated parameters included lung and blood metal accumulation, oxidative stress markers (malondialdehyde, nitric oxide), antioxidant defenses (superoxide dismutase, catalase, reduced glutathione, glutathione peroxidase), inflammatory cytokines (interleukin 6 [IL-6], tumor necrosis factor alpha [TNF-α]), transcription factors (nuclear factor erythroid 2-related factor 2 [Nrf2], nuclear factor kappa B [NF-κB]), caspase-3, hematological parameters, and lung histopathology. Multivariate analyses were applied to integrate biomarker patterns.

**Results:**

HMM exposure significantly increased tissue metal levels, oxidative stress, inflammatory mediators, and caspase-3 activity, while suppressing antioxidant defenses and disrupting hematological parameters and lung architecture. PA co-treatment reversed these changes in a dose-dependent manner. At 1500 mg/kg, PA reduced blood metal levels by > 90%, normalized the oxidative-antioxidant balance, decreased IL-6 and TNF-α by 60–70%, suppressed caspase-3 activity by 90%, restored hematological indices, and ameliorated lung damage. Principal component and hierarchical cluster analyses clearly separated the HMM-exposed groups from the PA-treated groups based on their distinct oxidative, inflammatory, and protective profiles.

**Conclusions:**

PA protects against HMM-induced toxicity by reducing metal bioavailability – potentially via chelation and/or altered absorption or excretion – coupled with Nrf2 activation and NF-κB/caspase-3 suppression. These findings suggest its potential as an adjunct therapy for heavy metalinduced pathologies.

## Introduction

Heavy metal pollution is a significant global health threat, particularly in regions with intense industrial activity and inadequate waste management (Yu et al. 2025). Because heavy metals are non-biodegradable and stable, they bioaccumulate long-term within the food chain and biological systems. Chronic exposure to metals such as lead (Pb), cadmium (Cd), and arsenic (As) is linked to various adverse health effects, including respiratory dysfunction, hematological disorders, immune system impairment, and carcinogenesis (Motta et al. 2025). In real-world settings, organisms are typically exposed to heavy metal mixtures (HMMs) rather than single metals in isolation; these mixtures often exhibit additive or synergistic toxicity (Hou et al. 2025). In polluted environments, Pb, Cd, and As frequently co-occur, making mixture toxicity models ecologically relevant (Kravchenko et al. 2025). Metal mixtures exacerbate oxidative stress, inflammation, and tissue injury more severely than individual metals (Hou et al. 2025). These mixtures induce toxicity primarily by generating reactive oxygen species (ROS) through electron transport chain disruption, sulfhydryl group binding, and antioxidant depletion, which leads to lipid peroxidation, protein oxidation, and DNA damage (Jomova et al. 2025).

The lung is a primary target of heavy metal toxicity via inhalational and systemic routes (Jin et al. 2025). Accumulated metals disrupt redox homeostasis, promoting ROS and nitric oxide formation, which results in oxidative damage and structural impairment. Exposure to HMM is associated with alveolar epithelial damage, pulmonary edema, interstitial inflammation, and progression toward chronic pathologies such as fibrosis (Jomova et al. 2025). This structural damage is often initiated by metal-induced vascular permeability and the subsequent influx of inflammatory mediators (Fatima et al. 2025). Metal-induced oxidative stress activates nuclear factor kappa B (NF-κB) signaling, driving the production of proinflammatory cytokines (interleukin 6 [IL-6], tumor necrosis factor alpha [TNF-α]), while suppressing nuclear factor erythroid 2-related factor 2 (Nrf2)-mediated antioxidant defenses (super-oxide dismutase [SOD], catalase [CAT], glutathione peroxidase [GPx]). These combined effects trigger caspase-3-dependent apoptosis (Barber et al. 2023; Kannan et al. 2025), leading to cell death and structural lung impairment (Kannan et al. 2025).

Beyond pulmonary damage, HMM exposure causes systemic hematological disturbances – such as anemia, reduced red blood cell (RBC) counts, decreased hemoglobin (Hb) levels, and altered platelet counts – and immune dysregulation, including leukocytosis, neutrophilia, and lymphopenia (Yasin and Salih 2025). Pb and Cd severely impact erythropoiesis by inhibiting key enzymes in Hb synthesis and increasing the oxidative fragility of the RBC membrane, resulting in anemia (Yuan et al. 2025). Furthermore, HMM exposure triggers an acute immunological shift characterized by leukocytosis and a pro-inflammatory differential leukocyte pattern with elevated neutrophils and monocytes, signaling systemic stress and an increased inflammatory burden (Sangubotla et al. 2025).

Current heavy metal poisoning management relies primarily on synthetic chelators, such as ethylenediaminetetraacetic acid (EDTA) (Fulgenzi et al. 2020). However, these agents have limitations, including poor membrane permeability, essential element depletion, and potential metal redistribution to vulnerable organs (Velugolu and Kumar 2025). These limitations necessitate exploring natural alternatives with dual chelating and antioxidant/anti-inflammatory properties (Singh and Sharma 2020). Ideal phytotherapeutic candidates should have dual actions: 1) chelation (metallo-remediation) to reduce the initial metal burden and 2) antioxidant/anti-inflammatory capacity to scavenge residual ROS and restore the Nrf2/NF-κB balance (Hashim et al. 2024). Furthermore, the application of advanced multivariate statistical analyses to decipher the interrelationships between these different levels of response remains limited in the existing literature (Garazhian et al. 2020).

*Prosopis africana* (PA) is a West African leguminous plant with an established history in traditional medicine (Aguirre-Loredo 2025). PA contains flavonoids, tannins, polyphenols, and alkaloids with documented antioxidant, anti-inflammatory, and metal-chelating properties (Zhong et al. 2022). Acute and sub-chronic toxicity studies indicate that PA has a relatively favorable safety profile (Obode et al. 2021). PA has been shown to demonstrate protective effects against heavy metal toxicity in neural and reproductive tissues by activating Nrf2 and reducing oxidative stress (Orisakwe et al. 2024). However, there is a lack of multivariate analyses exploring the protective effect of PA against HMMs in lung tissue across structural, biochemical, inflammatory, apoptotic, histological, and hematological endpoints (Garazhian et al. 2020). Given its phytochemical profile and established protective effects in other organ systems, PA represents a sustainable candidate for therapeutic intervention in heavy metal-induced toxicity (Botle et al. 2025; Yusoff et al. 2025).

Therefore, this study assessed metal bioaccumulation, the oxidative-antioxidant balance, inflammatory and apoptotic signaling, hematological profiles, and lung histopathology, while employing multivariate statistical analyses to integrate biomarker patterns.

We hypothesized that PA extract would dose-dependently reverse HMM-induced bioaccumulation, oxidative/inflammatory/apoptotic insults, hematologic disruption, and lung injury, thereby shifting biomarker and histological profiles toward those of the control group.

## Materials and methods

### Animal husbandry

Adult male Sprague-Dawley rats (*n* = 25; 180–200 g) were obtained from the University of Port Harcourt Animal House, Nigeria. The animals were housed in polypropylene cages with wood shavings and acclimatized for 14 days under standard conditions (12-h light/dark cycle, 25 ± 2°C, 50 ± 10% humidity) with ad libitum access to food and water (Ferrara et al. 2022).

### Ethical approval

All procedures were approved by the University of Port Harcourt Animal Use and Care Committee (Protocol No. UPH/CEREMAD/REC/MM73/014) and complied with the ARRIVE guidelines and the NIH Guide for the Care and Use of Laboratory Animals (Percie du Sert et al. 2020).

### HMM preparation

Analytical-grade salts – PbCl_2_ (99.999%), CdCl_2_ (99.995%), and Na_2_AsO_3_ (≥ 90%) – were purchased from Sigma-Aldrich (St. Louis, MO, USA). Each salt (1 g) was dissolved in 100 ml of deionized water and stored at 4°C until analysis (Ozoani et al. 2025).

### Plant material collection and extract preparation

Mature PA pods were collected in January 2021 from Nsukka, Enugu State, Nigeria. The specimens were botanically verified at the International Center for Ethnomedicine and Drug Discovery, University of Nigeria, Nsukka, where a voucher specimen (No. INTERCEDDO/033) was deposited. The plant collection complied with local biodiversity regulations without requiring special permits. The pods were sorted, washed, and sun-dried to constant weight. The dried pods were pulverized and homogenized using a mechanical grinder. Powdered material (100 g) was macerated in 1 l of methanol for 48 h with periodic shaking, filtered through Whatman No. 1 filter paper, concentrated by rotary evaporation, and oven-dried at 40°C for 48 h. The resulting dry methanolic extract was stored in an airtight container at 4°C until use (Ozoani et al. 2025). Although this study omitted batch-specific quantitative phytochemical profiling (e.g., total phenolic and flavonoid content), the phytochemical profile of PA methanolic extracts – including flavonoids, tannins, polyphenols, and alkaloids – is well-documented (Obode et al. 2021; Zhong et al. 2022), providing a basis for the mechanistic interpretations presented herein. However, future studies should include liquid chromatography–tandem mass spectrometry (LC–MS/MS)-guided batch characterization to improve reproducibility.

### Experimental design

After the 14-day acclimatization, the rats were randomly allocated into five groups (*n* = 5 per group) and treated daily via oral gavage for 60 days:

- Group 1 (Control): received deionized water.
- Group 2 (HMM only): received an HMM consisting of PbCl_2_ (20 mg/kg body weight [BW]), CdCl_2_ (1.61 mg/kg BW), and Na_2_AsO_3_ (10.0 mg/kg BW) (Ozoani et al. 2025).
- Group 3: received HMM plus PA extract at 500 mg/kg BW.
- Group 4: received HMM plus PA extract at 1000 mg/kg BW.
- Group 5: received HMM plus PA extract at 1500 mg/kg BW.

The dose range of 500–1500 mg/kg was selected based on the established acute oral toxicity profile of PA (median lethal dose, LD_50_ > 5000 mg/kg in rodents) (Obode et al. 2021), providing a safety margin exceeding 3-fold at the highest dose, and on dose ranges employed in comparable published models (Ozoani et al. 2025). The animals were observed daily throughout the 60-day treatment period for clinical signs of toxicity, including behavioral changes, coat condition, posture, and BW trajectory. No overt signs of toxicity or mortality occurred in any PA-treated group.

### Sample collection and tissue preparation

On day 60, the animals were weighed and euthanized via an intraperitoneal injection of pentobarbital (90 mg/kg BW). Blood (~3 ml) was collected via retro-orbital puncture under anesthesia. The samples were split into two fractions: one in a plain tube for heavy metal analysis, and the other in an EDTA vacutainer for hematological assessment (Okolo et al. 2020). After euthanasia, the lungs were excised, weighed to determine relative organ weights, and rinsed twice in ice-cold saline buffer (20 mM Tris-HCl, 0.14 M NaCl, pH 7.4). Each lung was divided into portions for histological and biochemical analyses. Lung tissue was fixed in 10% neutral-buffered formalin, processed routinely, embedded in paraffin, sectioned (3–5 µm), and stained with hematoxylin and eosin. A certified histopathologist, blinded to group allocation, examined the sections using a Leica DM6B microscope (Leica Microsystems, Germany) at ×100 and ×400 magnifications (Slaoui et al. 2017).

### Tissue homogenization and biochemical assays

Tissue was homogenized (10%, w/v) in ice-cold 50 mM Tris-HCl buffer (pH 7.4), sonicated, and centrifuged at 3000 × g for 15 min at 4°C. The resulting supernatants were aliquoted and stored at –20°C in cryovials for subsequent biochemical and heavy metal analyses (Hao et al. 2025).

### Measurement of lung weights and calculation of relative organ weight

Excised lungs were rinsed in saline buffer (pH 7.4), blotted dry, and weighed to calculate relative lung weight:


$$\begin{array}{l} \text{Relative lung weight}(\%)=\\ =(\text{Lung weight}(\text{g})//\text{Animal BW at day}60(\text{g})\times 100 \end{array}$$

### Heavy metal analysis

Lung tissue (~1 g) was digested in 2 ml of 0.1 M HCl and 6 ml of concentrated HNO_3_ using acid-washed glassware. The mixture was heated at 105°C until clear, and then filtered through Whatman No. 42 filter paper. The filtrate was diluted to 25 ml with deionized water in a volumetric flask. The concentrations of Pb, Cd, and As were determined using a flame atomic absorption spectrophotometer (FAAS, Model S4 71096). All measurements were conducted in triplicate following Cobbina et al. (2015). Recovery rates ranged from 94.5% to 100%, with relative standard deviations (SDs) below 4% across replicates. The limits of detection were 0.001 mg/kg for both As and Cd, and 0.01 mg/kg for Pb. The corresponding limits of quantification were 0.0033 mg/kg for As and Cd, and 0.033 mg/kg for Pb, as reported by Messarah et al. (2012).

## Assessment of oxidative stress markers

Lipid peroxidation levels in the lung tissues were quantified by measuring malondialdehyde (MDA) via the thiobarbituric acid reactive substances method, with absorbance read at 532 nm (Ozoani et al. 2024). NO concentrations were evaluated spectrophotometrically at 540 nm, following Goshi et al. (2019). SOD activity was assessed by its ability to inhibit pyrogallol auto-oxidation, with absorbance changes recorded every 30 s over 3 min at 420 nm (Bizoñ et al. 2022). Reduced glutathione (GSH) levels and GPx activity were determined at 450 and 340 nm, respectively, using methods adapted from Jollow et al. (1974). CAT activity was measured using a modified method from Lewden et al. (1996), which is based on the enzymatic decomposition of hydrogen peroxide, monitored at 240 nm with a spectrophotometer. All assays were performed in triplicate. Results were expressed in µM/mg for MDA, µM for NO, and µg/mg for GSH, SOD, CAT, and GPx.

### Pro-inflammatory, apoptotic, and transcriptional marker analysis

Levels of TNF-α, IL-6, caspase-3 (apoptotic marker), NF-κB, and Nrf2 were quantified in lung tissue homogenates using commercial enzyme-linked immunosorbent assay (ELISA) kits validated for rat samples (Elab Science Biotechnology Co., Wuhan, Hubei, People’s Republic of China), following the manufacturers’ instructions. Absorbance was measured using a microplate reader, and concentrations were calculated from standard curves. Results were expressed in pg/ml for IL-6, TNF-α, and Nrf2, and ng/ml for NF-κB and caspase-3.

### Hematological analyses

Packed cell volume (PCV) was determined using the microhematocrit technique (Farooq et al. 2023). Hb concentration was measured colorimetrically using the cyanmethemoglobin method (Arnaud et al. 2017). Platelets were counted under oil immersion (×1000) across 10 fields and multiplied by 20,000 for total count estimation (Akinniyi et al. 2025). Erythrocyte and total leukocyte counts were performed using a Neubauer hemocytometer with Natt-Herrick solution (1 : 200 dilution) as the diluent (Orakpoghenor et al. 2021). For differential leukocyte counts, blood smears were prepared and stained via the Giemsa technique, and cells were enumerated using the battlement track counting method (Ugwuanyi et al. 2025). Results were expressed as percentages for differential counts and as absolute numbers (× 10^9^/l) for total counts.

### Statistical analysis

Data analysis was performed using GraphPad Prism version 9.0.0 (GraphPad Software, San Diego, CA, USA), with results expressed as mean ± SD (*n* = 5). Group comparisons were made using a one-way analysis of variance followed by Tukey’s *post hoc* test for pairwise differences, with *p* < 0.05 indicating statistical significance. Multivariate data exploration was conducted using XLSTAT version 2019 (Addinsoft, Paris, France). To identify major trends and correlations among lung and blood parameters, principal component analysis (PCA) and cluster analysis were performed. Principal axis factoring was used for extraction, and varimax rotation optimized the interpretability of results. Relationships among hematological and biochemical indices were visualized using a hierarchical cluster heatmap. Before analysis, all parameters were normalized through *Z*-score transformation to eliminate scaling bias and ensure a uniform data distribution using the formula:

$$Z=\frac{{\text{X}}_{\text{i}}-\mu }{\sigma },$$

where X_i_ is the individual observed value, µ is the group mean, and σ is the SD.

*Z*-scores for protective variables (SOD, CAT, GSH, GPx, Nrf2) were inverted (by multiplying by –1) so higher values uniformly represented pathological states across all parameters on the heatmap. Hierarchical clustering was performed using Euclidean distance and Ward’s linkage method.

## Results

### Heavy metal bioaccumulation in lung tissue

The concentrations of Pb, Cd, and As across all experimental groups are shown in Figure 1. Compared with controls, HMM exposure significantly elevated lung Pb, Cd, and As levels by 1998-, 250-, and 252-fold, respectively (*p* ≤ 0.001; Figure 1). PA co-treatment dose-dependently reduced this accumulation: the 1500 mg/kg dose decreased Pb, Cd, and As levels by 59%, 41%, and 40%, respectively, relative to the HMM-only group (*p* ≤ 0.001).

**Figure 1:**
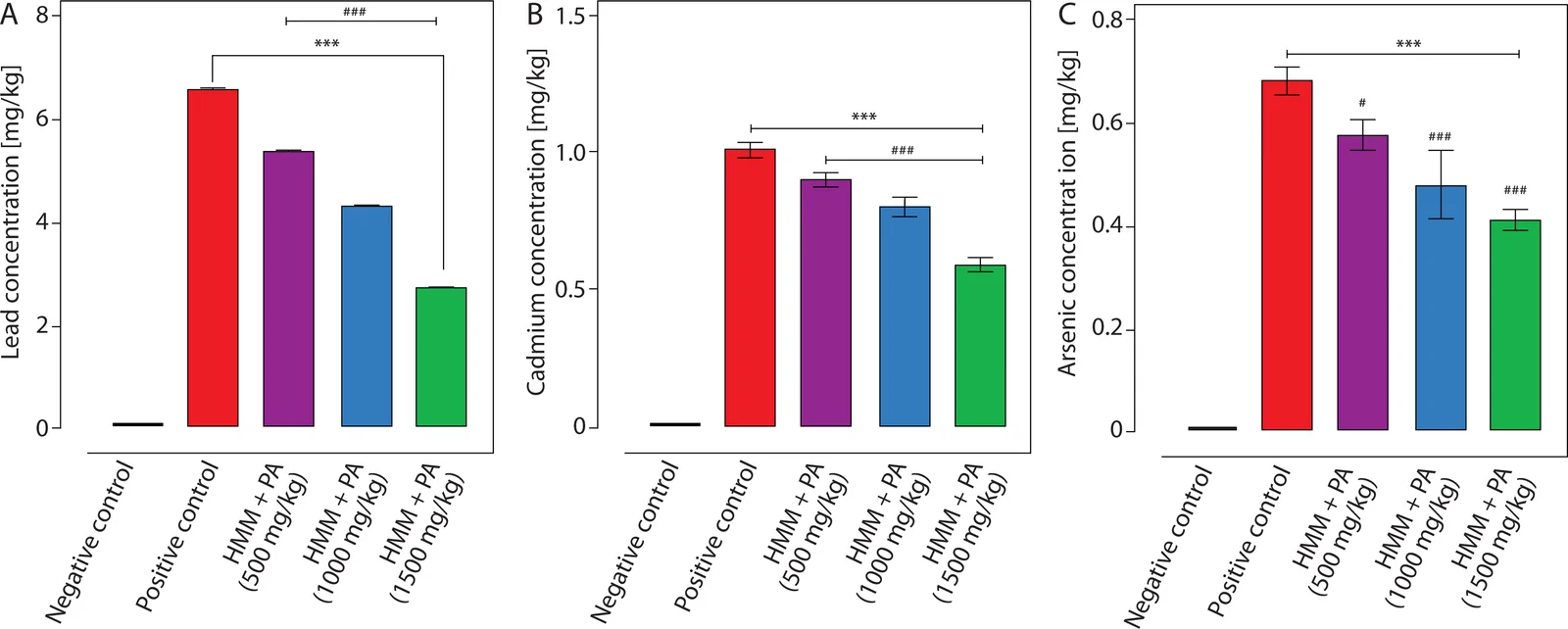
Tissue concentrations of Pb, Cd, and As (mean ± SD) in the lungs of experimental groups: negative control, positive control (HMM only), and HMM + PA at 500, 1000, and 1500 mg/kg, *n* = 5. Statistical comparisons were made by one-way ANOVA analysis of variance with Tukey’s post hoc test. ***p < 0.001 (relative to normal control); ^#^*p* < 0.05, ^###^*p* < 0.001 (relative to positive control). ANOVA – analysis of variance, As – arsenic, Cd – cadmium, HMM – heavy metal mixture, PA – *Prosopis africana*, Pb – lead, SD – standard deviation

### Heavy metal concentrations in blood

Figure 2 shows the blood concentrations (mean ± SD) of Pb, Cd, and As across experimental groups, which mirrored lung accumulation patterns. HMM exposure increased blood Pb, Cd, and As levels by 94-, 40-, and 140-fold, respectively (*p* ≤ 0.001). PA treatment dose-dependently reduced this blood metal burden: the 1500 mg/kg dose achieved reductions of ≥ 90% for Pb and Cd, and 86% for As, restoring concentrations near control values (*p* ≤ 0.001).

**Figure 2:**
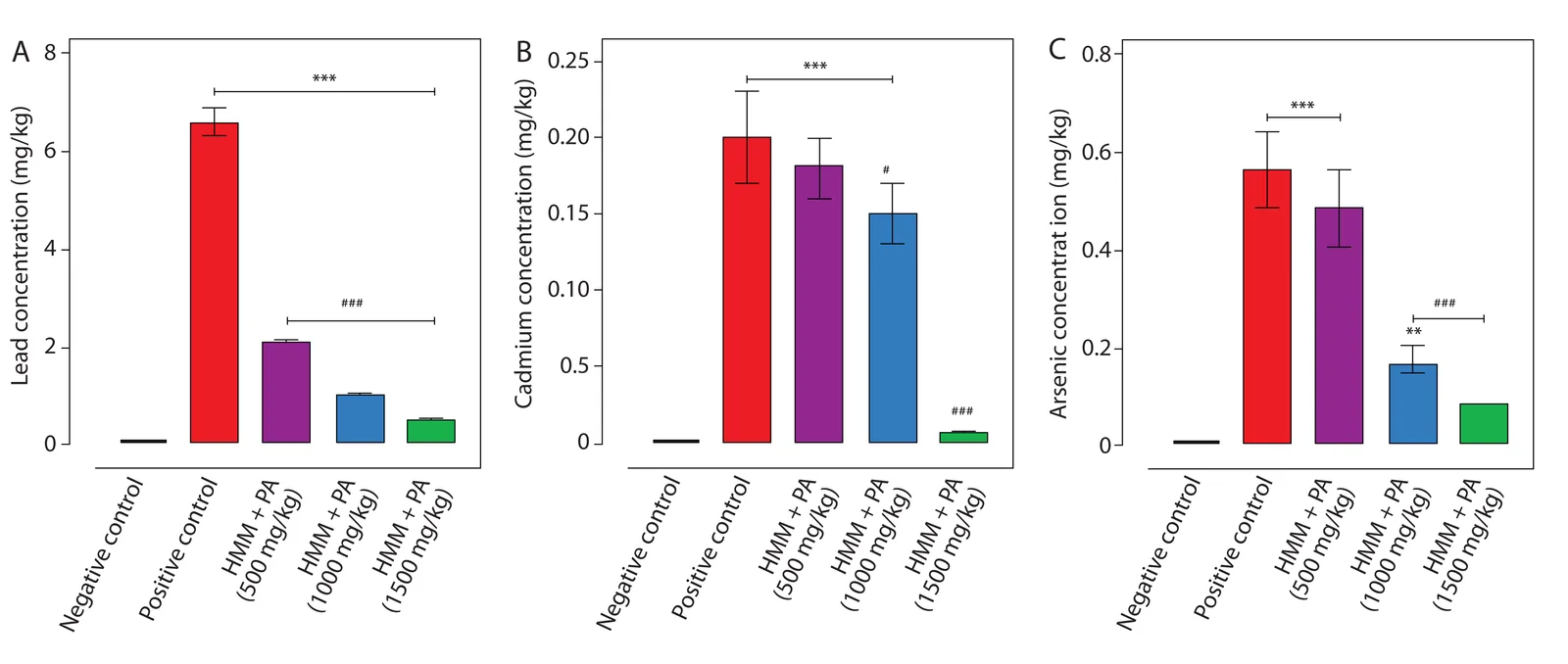
Blood concentrations of Pb, Cd, and As across experimental groups (**A, B**, and **C**, respectively). Values are presented as mean ± SD (*n* = 5 per group). **p ≤ 0.01; ***p ≤ 0.001 (relative to negative control), ^#^*p* ≤ 0.01, ^###^*p* ≤ 0.001 (relative to positive control). As – arsenic, Cd – cadmium, Pb – lead, SD – standard deviation

### Lung morphometry

The absolute and relative lung weights (mean ± SD) across all experimental groups are shown in Figure 3. HMM exposure increased absolute and relative lung weights by 31% and 47%, respectively, though the changes were not statistically significant (*p* > 0.05; Figure 3). PA treatment produced dose-dependent trends toward normalization without achieving statistical significance.

**Figure 3:**
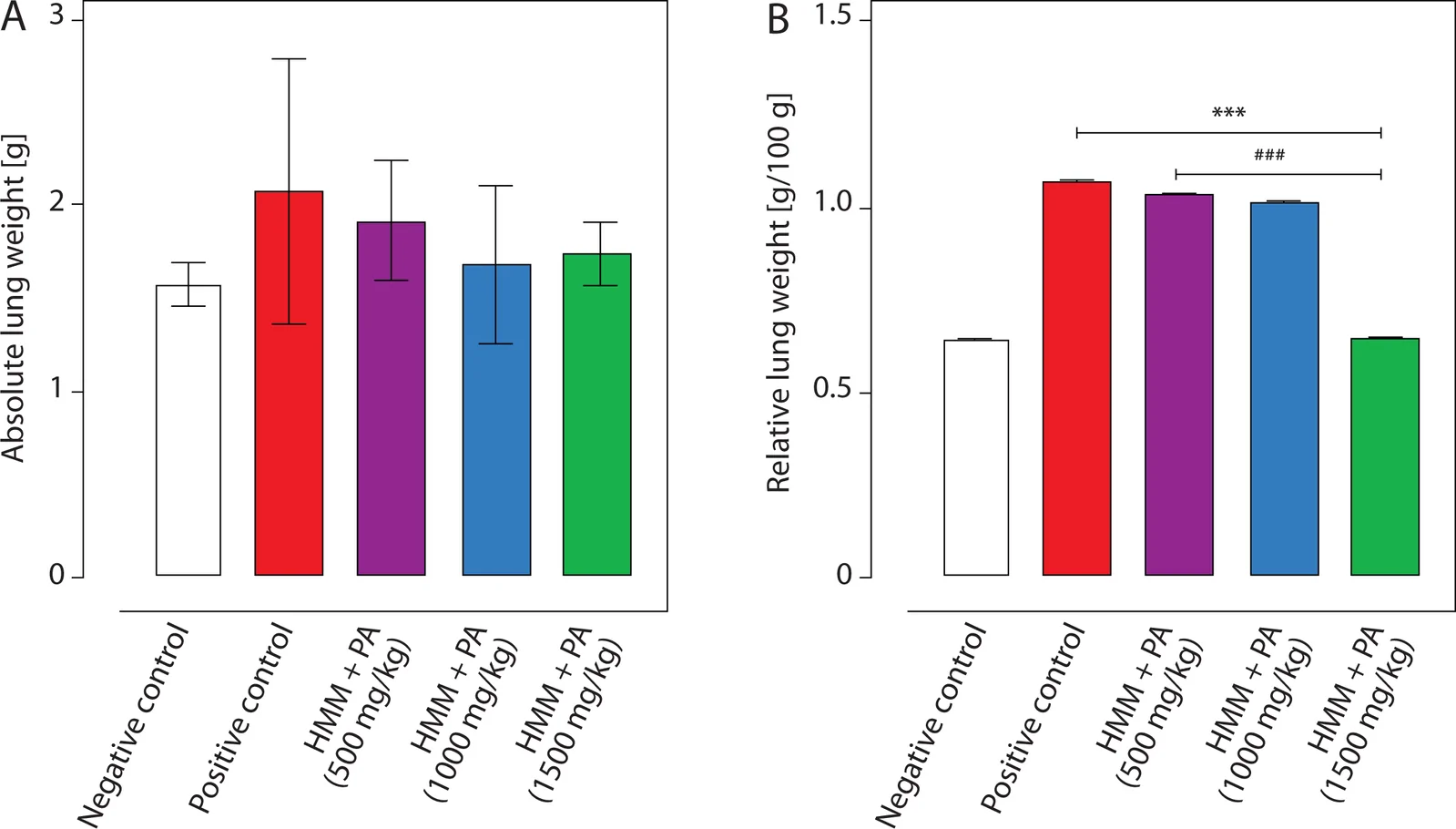
(**A**) Absolute [g] and (**B**) relative (organ-to-body weight ratio) lung weights (mean ± SD) in negative control, positive control (HMM only), and HMM + PA at 500, 1000, and 1500 mg/kg, *n* = 5. Percentage changes are calculated relative to the positive control group. No group differences were statistically significant by one-way ANOVA (*p* > 0.05). ANOVA – analysis of variance, HMM – heavy metal mixture, PA – *Prosopis africana*, SD – standard deviation

### Oxidative stress and antioxidant enzyme activity

HMM exposure significantly induced oxidative stress (MDA, NO) while suppressing antioxidant enzyme (SOD, CAT, GSH, GPx) activities in lung tissue, whereas administration of PA extract dose-dependently induced partial rescue (Figure 4).

**Figure 4:**
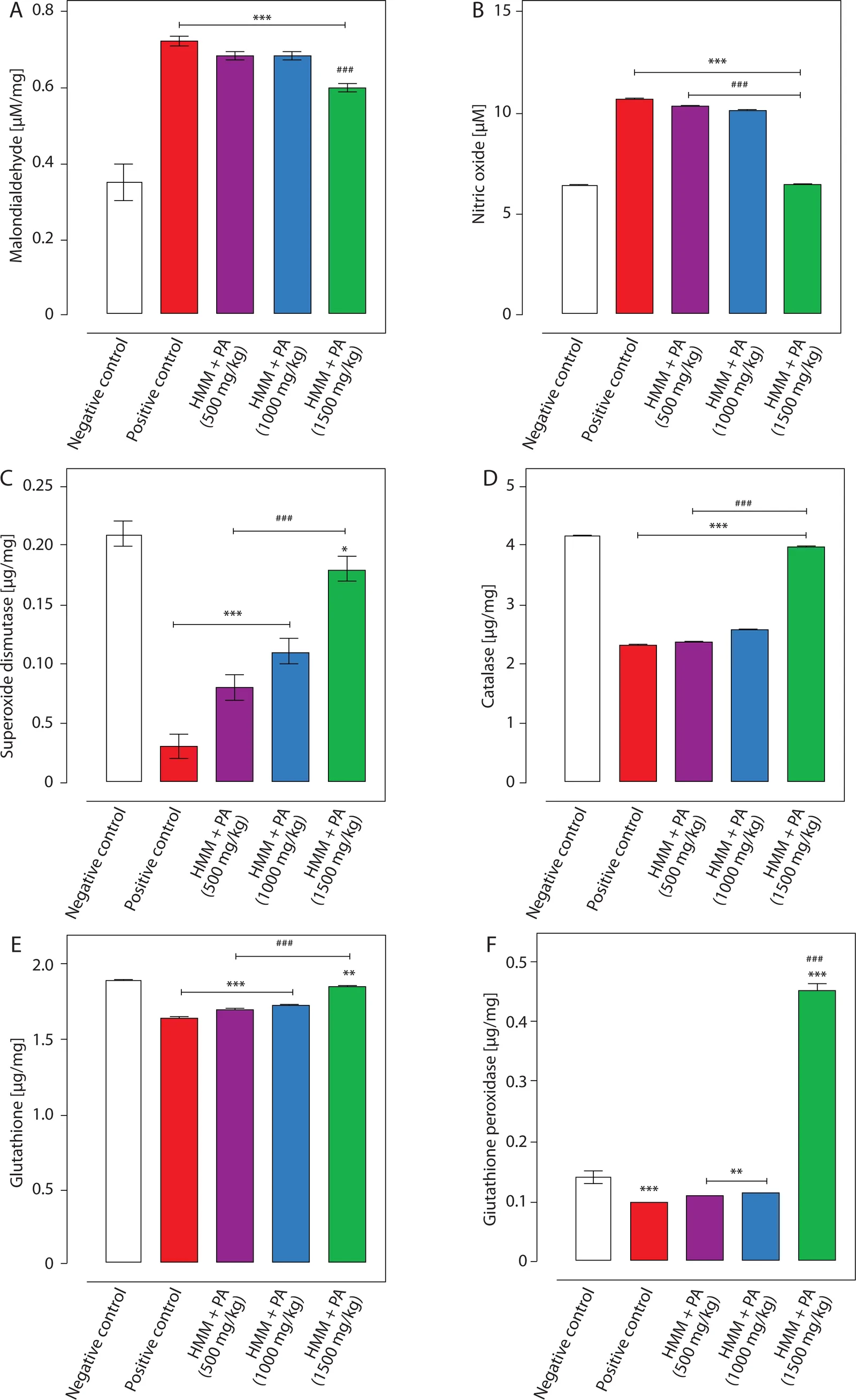
Effects of PA on oxidative stress and antioxidant enzyme activity. Values are presented as mean ± SD, *n* = 5 per group. **p < 0.01, ***p < 0.001 (vs. negative control), ^###^*p* < 0.001 (vs. positive control). Mean differences shown reflect post hoc pairwise comparisons (Tukey’s test) via one-way ANOVA. ANOVA – analysis of variance, CAT – catalase, GPx – glutathione peroxidase, GSH – reduced glutathione, MDA – malondialdehyde, NO – nitric oxide, PA – *Prosopis africana*, SD – standard deviation, SOD – superoxide dismutase

Specifically, HMM exposure caused a 2.1- and 1.7-fold increase in MDA and NO levels, respectively, alongside substantial decreases in SOD, CAT, GSH, and GPx activities by 86%, 45%, 16%, and 29%, respectively, compared with controls (*p* ≤ 0.001; Figure 4).

PA co-treatment dose-dependently restored this oxidative-antioxidant balance. At 1500 mg/kg, MDA and NO levels decreased by 17% and 38% relative to the HMM-only group (*p* ≤ 0.001). Concurrently, antioxidant activities restored substantially: SOD increased 6-fold, CAT 1.7-fold, GSH 1.2-fold, and GPx 4.5-fold compared with the HMM-only group (*p* ≤ 0.001). The highest PA dose nearly normalized all parameters to control levels.

### Inflammatory, transcriptional, and apoptotic responses

As shown in Table 1, HMM exposure provoked strong inflammatory, transcriptional, and apoptotic responses. HMM significantly elevated pro-inflammatory cytokines (IL-6: 7.0-fold; TNF-α: 4.1-fold), NF-κB (12.2-fold), and caspase-3 (12-fold) compared with controls (*p* ≤ 0.001; Table 1). Notably, Nrf2 protein expression surged 39-fold above control levels (Table 1), reflecting a compensatory transcriptional stress response to an overwhelming oxidative burden. Despite this overexpression, antioxidant enzyme activities were profoundly suppressed (SOD: –86%; CAT: –45%; GSH: –16%; GPx: –29%; Figure 4), indicating that the elevated Nrf2 protein was functionally insufficient. This aligns with metal-induced impairment of Nrf2 nuclear translocation and antioxidant response element (ARE)-binding activity.

**Table 1:** Lung tissue levels of IL-6, TNF-α, NF-κB, Nrf2, and caspase-3 across treatment groups

Groups	IL-6 [pg/ml]	TNF-α [pg/ml]	NF-κB [ng/ml]	Nrf2 [pg/ml]	Caspase-3 [ng/ml]
Negative control	51 ± 0.35	40 ± 0.35	1.37 ± 0.04	0.07 ± 0.00	12 ± 0.28
Positive control	358 ± 3.53^***^	165 ± 6.36^***^	16.7 ± 0.42^***^	2.71 ± 0.02^***^	144 ± 1.41^***^
HMM + PA (500 mg/kg)	299 ± 4.26^***,###^	162 ± 1.41^***^	12.3 ± 0.28^***,###^	2.56 ± 0.02^***,###^	100 ± 1.27^***,###^
HMM + PA (1000 mg/kg)	200 ± 2.82^***,###^	113 ± 0.91^***,###^	5.42 ± 0.01^***,###^	2.70 ± 0.02^***,###^	39 ± 0.56^***,###^
HMM + PA (1500 mg/kg)	127 ± 1.41^***,###^	52 ± 2.40^###^	4.13 ± 0.01^***,###^	0.14 ± 0.01^***,###^	14 ± 0.28^*,###^

Values are presented as mean ± SD (*n* = 5 per group).

* *p* ≤ 0.05 (vs. negative control);

*** *p* ≤ 0.001 (vs. negative control);

### *p* ≤ 0.001 (vs. positive control).

IL-6 – interleukin-6, NF-κB – nuclear factor kappa B, Nrf2 – nuclear factor erythroid 2-related factor 2, SD – standard deviation, TNF-α – tumor necrosis factor alpha

PA treatment progressively attenuated these responses in a dose-dependent manner. At 1500 mg/kg, IL-6 and TNF-α decreased by 64.5% and 68.5%, respectively, NF-κB by 75.3%, and caspase-3 by 90.3% relative to the HMM-only group (*p* ≤ 0.001). Specifically, Nrf2 expression was restored almost fully (95.2% normalization), approaching control levels.

### Lung histopathology

Histopathological assessment of the lung sections revealed distinct alterations among the treatment groups (Figure 5). Control lungs exhibited normal pulmonary architecture with intact bronchi, alveolar septa, and vessels. In contrast, HMM-exposed lungs showed marked interstitial and perivascular congestion accompanied by intraluminal blood casts (BCs), indicating inflammatory injury.

**Figure 5:**
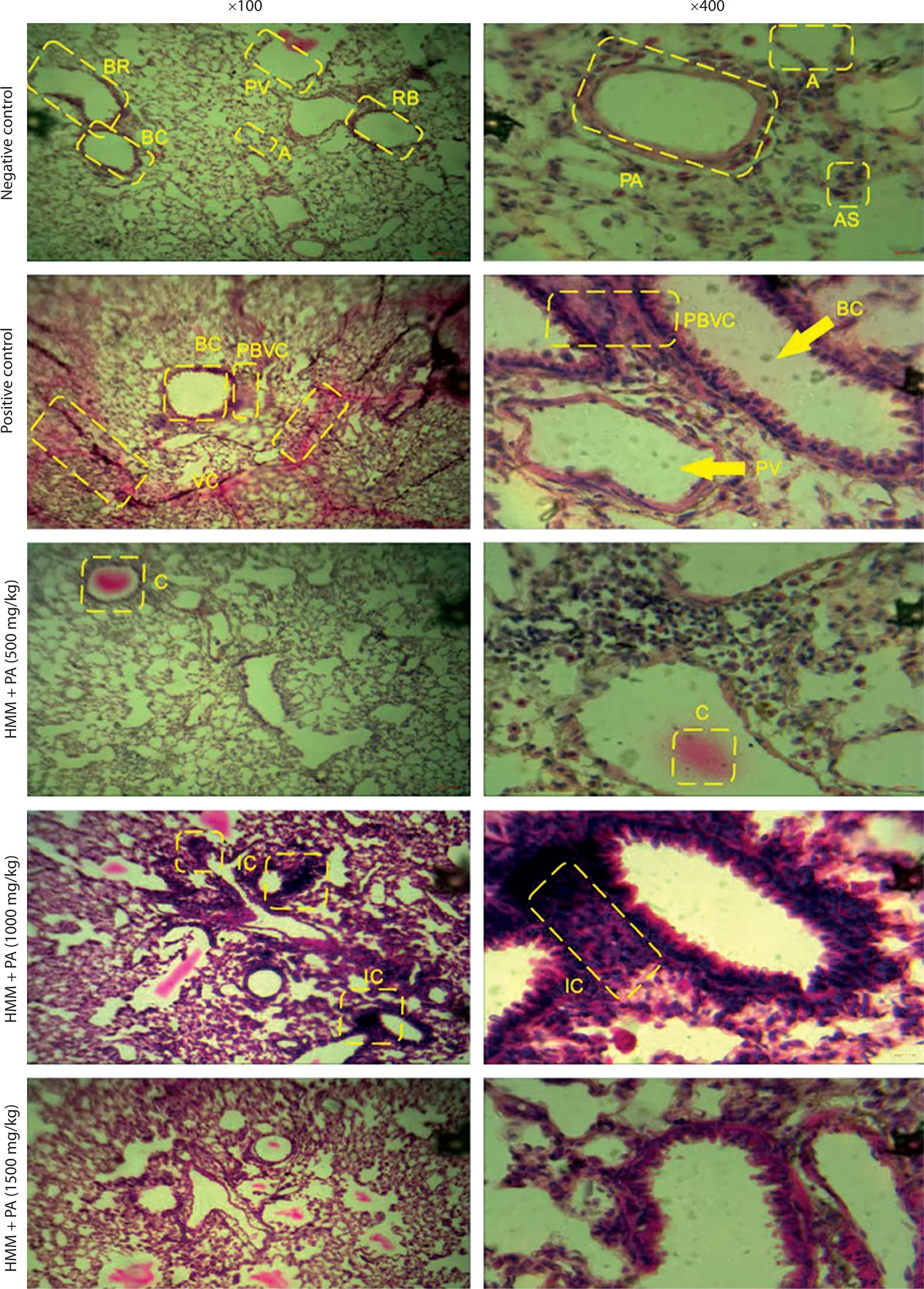
Representative photomicrographs of lung tissues showing the histopathological changes following PA treatment in heavy metal-exposed rats. Negative control (normal architecture): A – alveoli, AS – alveolar septum, BC – bronchiole, BR – bronchus, PV – pulmonary vein. Positive control: PBVC – interstitial and peribronchiolar vascular congestion. PA 500 mg/kg: moderate vascular congestion and blood casts. PA 1000 mg/kg: C – blood casts, VC – moderate perivascular congestion. PA 1500 mg/kg: restored architecture and minimal or no congestion. PA – *Prosopis africana*

Co-treatment with PA extract dose-dependently ameliorated these lesions. The 500 mg/kg and 1000 mg/kg groups exhibited cysts and inflammatory cell infiltration, suggesting partial protection at these doses. Remarkably, the 1500 mg/kg group displayed nearly normal pulmonary histoarchitecture with resolved vascular congestion and an absence of BCs, closely resembling control morphology. These findings indicate that PA extract restored heavy metal-induced pulmonary damage, particularly at the highest dose.

### Erythrocyte and platelet parameters

Table 2 shows the effects of HMM exposure on PCV, RBC, Hb, and platelets in PA-treated rats. HMM exposure significantly reduced PCV (by 25.5%), RBC (by 24.6%), and Hb (by 25.2%), while increasing platelet counts by 176% (*p* ≤ 0.001; Table 2). PA co-treatment dose-dependently reversed these alterations; the 1500 mg/kg dose nearly normalized all parameters. Specifically, PCV, RBC, and Hb recovered to 4.3%, 7.7%, and 3.2% of control values, respectively, while platelet counts decreased to near-baseline levels (62.5% reduction vs the HMM group; *p* ≤ 0.001).

**Table 2:** Hematological indices: PCV, RBC, Hb, and platelet count in HMM-induced rats

Groups	PCV (%)	RBC [×10^12^/l]	Hb [g/dl]	Platelet [×10^9^/l]
Negative control	47 ± 1.5	6.5 ± 0.25	15.5 ± 0.5	261 ± 40
Positive control	35 ± 3.0^***^	4.9 ± 0.47^**^	11.6 ± 1.0^***^	722 ± 57^***^
HMM + PA (500 mg/kg)	39 ± 1.0^**^	5.7 ± 0.46	13.0 ± 0.4^**^	547 ± 60^***,##^
HMM + PA (1000 mg/kg)	41 ± 2.5^*,#^	6.0 ± 0.12^#^	13.8 ± 0.8^*,#^	502 ± 26^***,###^
HMM + PA (1500 mg/kg)	45 ± 1.0^###^	6.0 ± 0.32^#^	15.0 ± 0.3^###^	271 ± 18^###^

Values are presented as mean ± SD (*n* = 5 per group).

* *p* ≤ 0.05,

** *p* ≤ 0.01,

*** *p* ≤ 0.001 (relative to negative control);

# *p* ≤ 0.05,

## *p* ≤ 0.01,

### *p* ≤ 0.001 (relative to positive control).

Hb – hemoglobin, HMM – heavy metal mixture, PCV – packed cell volume, PLT – platelet, RBC – red blood cell, SD – standard deviation

### Leukocyte profile

HMM exposure induced leukocytosis (132% increase vs. control; *p* ≤ 0.01), characterized by lymphopenia (19.1% decrease), neutrophilia (52.2% increase), and monocytosis (56.6% increase; Table 3). PA treatment progressively normalized these changes. At 1500 mg/kg, the total white blood cell (WBC) count returned to control levels (*p* ≤ 0.01 vs. HMM), the lymphocyte percentage increased to 70%, and the neutrophil and monocyte percentages decreased to baseline, reflecting the resolution of systemic inflammation.

**Table 3:** Total WBC count and differential leukocyte percentages across treatment groups

Groups	WBC [×10^9^/l]	L (%)	N (%)	M (%)	E (%)	B (%)
Negative control	6.9 ± 2.5	68 ± 2.3	23 ± 1.5	5.3 ± 0.6	3.7 ± 0.6	0
Positive control	16.0 ± 1.7^**^	55 ± 5.0^**^	35 ± 3.0***	8.3 ± 1.5^*^	2.7 ± 0.7	0
HMM + PA (500 mg/kg)	15.0 ± 2.8^**^	63 ± 0.6^#^	28 ± 2.0^#^	6.3 ± 1.2	2.7 ± 0.5	0
HMM + PA (1000 mg/kg)	12.0 ± 2.1	68 ± 3.5^##^	22 ± 2.0^###^	6.0 ± 1.0	3.3 ± 0.6	0
HMM + PA (1500 mg/kg)	6.8 ± 0.9^##^	70 ± 0.6^###^	22 ± 0.6^###^	5.3 ± 0.6^#^	3.3 ± 0.6	0

Values are presented as mean ± SD (*n* = 5 per group).

* *p* ≤ 0.05,

** *p* ≤ 0.01 (vs. negative control);

# *p* ≤ 0.05,

## *p* ≤ 0.01,

### *p* ≤ 0.001 (vs. positive control).

B – basophils (%), E – eosinophils (%), L – lymphocytes (%), M – monocytes (%), N – neutrophils (%), SD – standard deviation, WBC – white blood cell (×10^9^/l)

### Correlation analysis of biomarkers

Pearson correlation analysis revealed strong associations between metal burden, oxidative stress, and inflammatory markers (Figure 6). In the lungs, Pb, Cd, and As levels positively correlated with MDA, NO, and inflammatory cytokines (*r* > 0.8) and negatively correlated with antioxidant enzymes and Nrf2 (*r* = –0.85 to –0.99). IL-6, TNF-α, and caspase-3 showed tight inter-correlations (*r* > 0.95), confirming coordinated oxidative-inflammatory responses.

**Figure 6:**
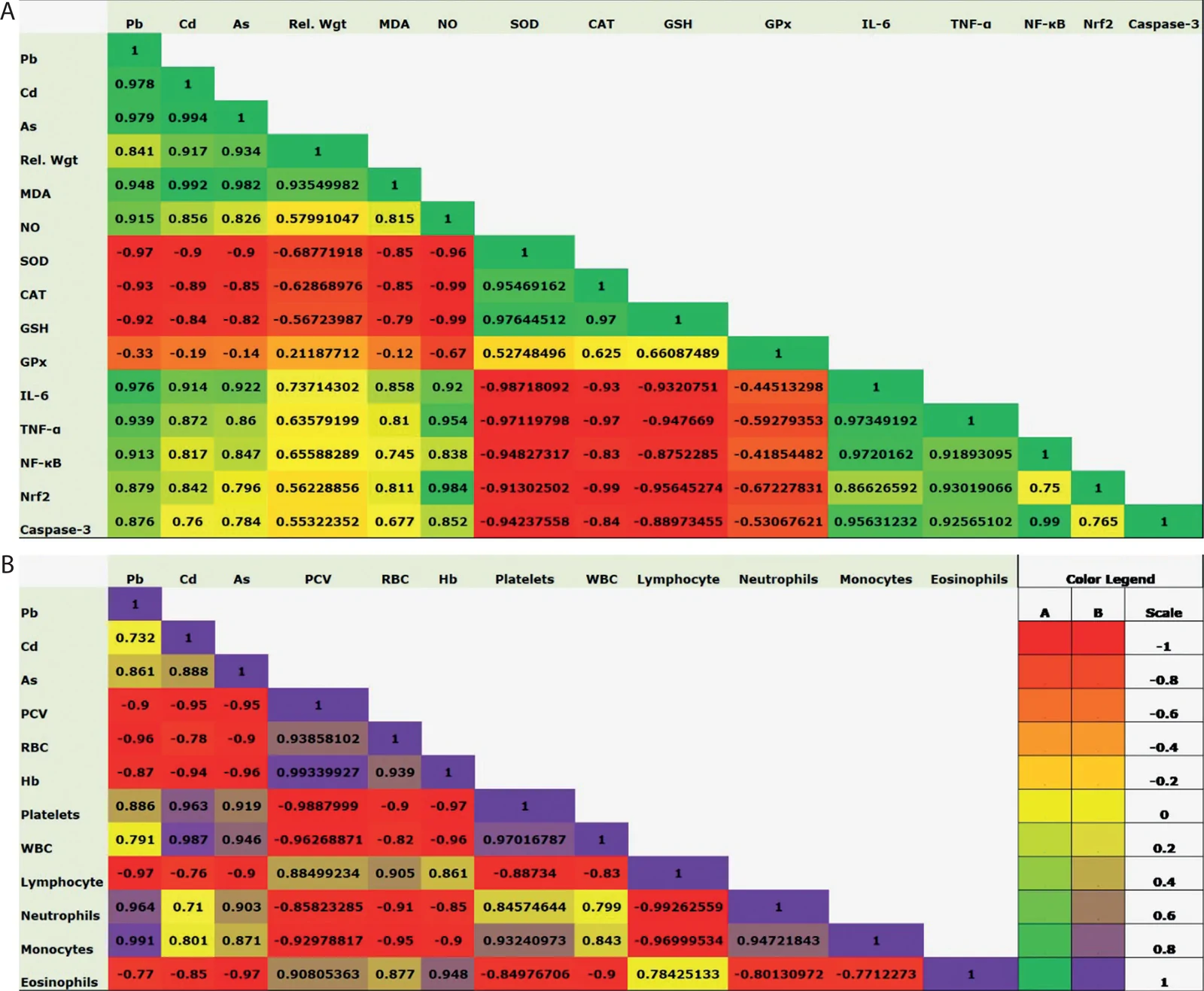
Intervariable Pearson correlation among heavy metals, oxidative stress markers, antioxidants, and hematological indices. *n* = 5 per group. All values are Pearson correlation coefficients (*r*). Positive correlations indicate direct association; negative correlations indicate inverse association. As – arsenic, CAT – catalase, Cd – cadmium, GPx – glutathione peroxidase, GSH – reduced glutathione, IL-6 – interleukin-6, MDA – malondialdehyde, NF-κB – nuclear factor kappa B, NO – nitric oxide, Nrf2 – nuclear factor erythroid 2-related factor 2, Pb – lead, Rel. Wgt – relative lung weight, SOD – superoxide dismutase, TNF-α – tumor necrosis factor alpha

In the blood, metal levels negatively correlated with erythrocyte parameters (PCV, RBC, Hb; *r* = –0.87 to –0.96) and positively correlated with platelet and WBC counts (*r* > 0.78). Platelet and WBC counts correlated positively with neutrophils and monocytes (*r* > 0.84), whereas lymphocytes showed inverse associations (*r* < –0.97), indicating systemic immune dysregulation. These patterns confirm that PA intervention restores homeostasis by modulating Nrf2/NF-κB signaling and reducing oxidative burden.

### PCA

PCA identified major variance patterns in the lung and blood datasets (Table 4; Figure 6). For lung parameters, first principal component (PC1) and second principal component (PC2) accounted for 83.84% and 12.10% of the total variance, respectively, while for the blood, they explained 90.33% and 5.98%. This confirms that the first two components effectively captured most treatment-induced variations.

**Table 4:** Eigenvalues, percentage, and cumulative variance of principal components for lung and blood datasets

Components	F1	F2	F3	F4
Lungs				
Eigenvalue	12.58	1.82	0.55	0.06
Variability (%)	83.84	12.1	3.64	0.41
Cumulative (%)	83.84	95.94	99.59	100
Blood				
Eigenvalue	10.84	0.72	0.28	0.16
Variability (%)	90.33	5.98	2.32	1.37
Cumulative (%)	90.33	96.31	98.63	100

F1 and F2 represent the first two principal components, explaining most of the variance among variables. Eigenvalues > 1 were considered significant.

The PCA biplot revealed distinct clustering of the experimental groups (Figure 7). HMM-exposed animals clustered along the negative PC1 axis, driven by high loadings of Pb, Cd, As, oxidative stress markers (MDA, NO), inflammatory cytokines (IL-6, TNF-α, NF-κB), and caspase-3. Conversely, the PA-treated groups aligned with the positive PC1 axis, associated with strong loadings of antioxidant enzymes (SOD, CAT, GSH, GPx) and Nrf2, reflecting an enhanced protective capacity.

**Figure 7:**
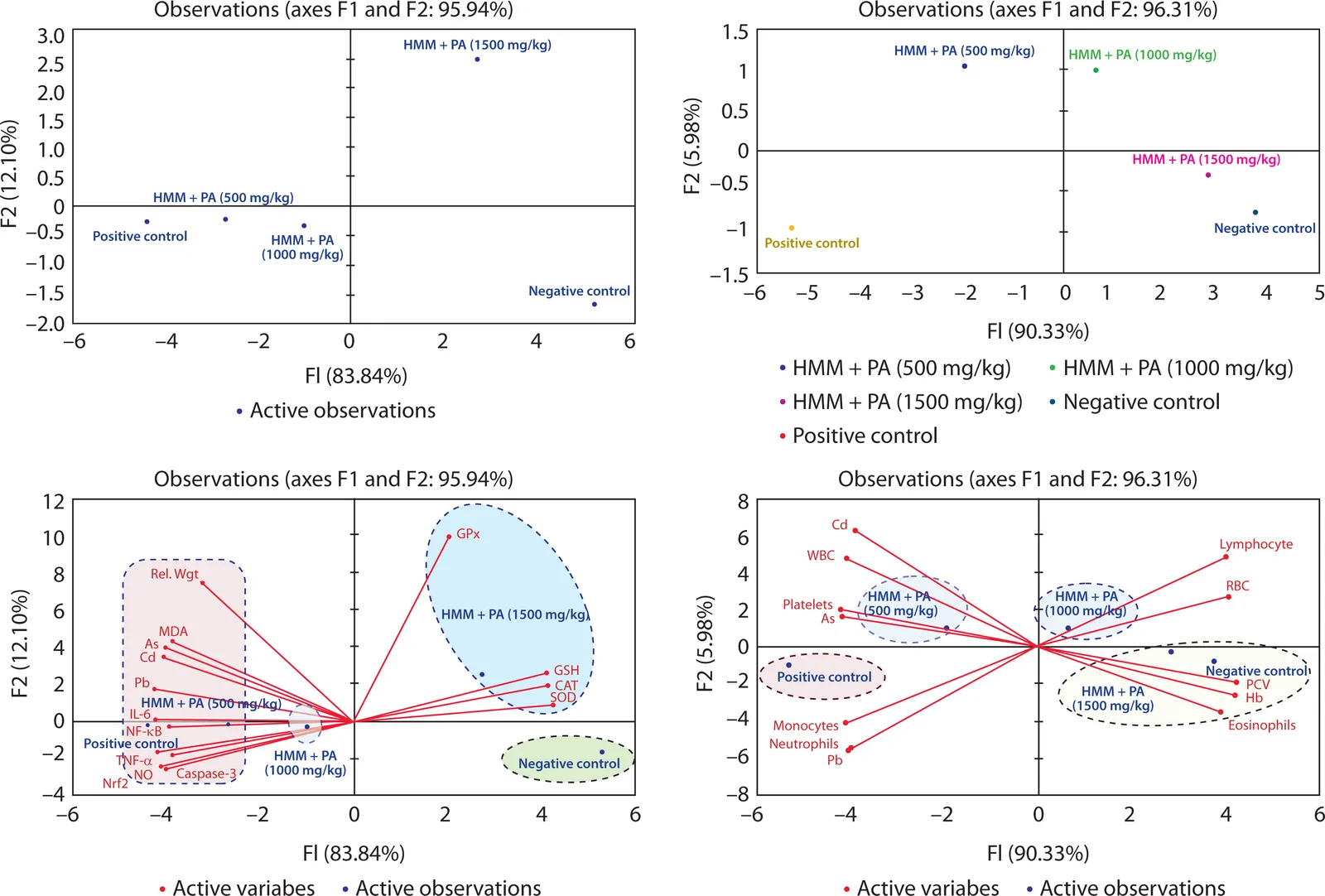
Principal component analysis plots showing the clustering and contribution of variables among treatment groups

For hematological parameters, PCV, RBC, Hb, and lymphocytes loaded positively on PC1, indicating their association with improved hematopoietic function, whereas WBC, neutrophils, and platelets loaded negatively, reflecting the inflammatory burden in HMM-exposed animals. The 1500 mg/kg group clustered closest to the controls in both datasets.

These patterns demonstrate that PA treatment systematically shifted the biochemical and hematological profiles from an oxidative-inflammatory state to a protective-homeostatic state, in a dose-dependent manner.

Panels A_1_ and A_2_ represent the observation loadings and biplot, respectively, for lung variables, while panels B_1_ and B_2_ represent the corresponding profiles for blood variables. The plots illustrate the distinct separation between control, heavy metal-exposed, and extracttreated groups based on the distribution of oxidative stress, inflammatory, antioxidant, and hematological markers.

### Hierarchical cluster analysis

Hierarchical cluster analysis (HCA) of *Z*-score-normalized data revealed clear separation between toxic and protected states (Figure 8). In lung tissue, the HMM-exposed group clustered distinctly due to an elevated metal burden (Pb, Cd, As), inflammatory markers (TNF-α, IL-6, NF-κB, caspase-3), and oxidative stress (MDA, NO), alongside suppressed antioxidant defenses (SOD, CAT, GSH, GPx, Nrf2). All PA-treated groups clustered separately from the HMM group, with the 1500 mg/kg dose clustering closest to the controls, confirming a dose-dependent reversal of the toxic phenotype.

**Figure 8:**
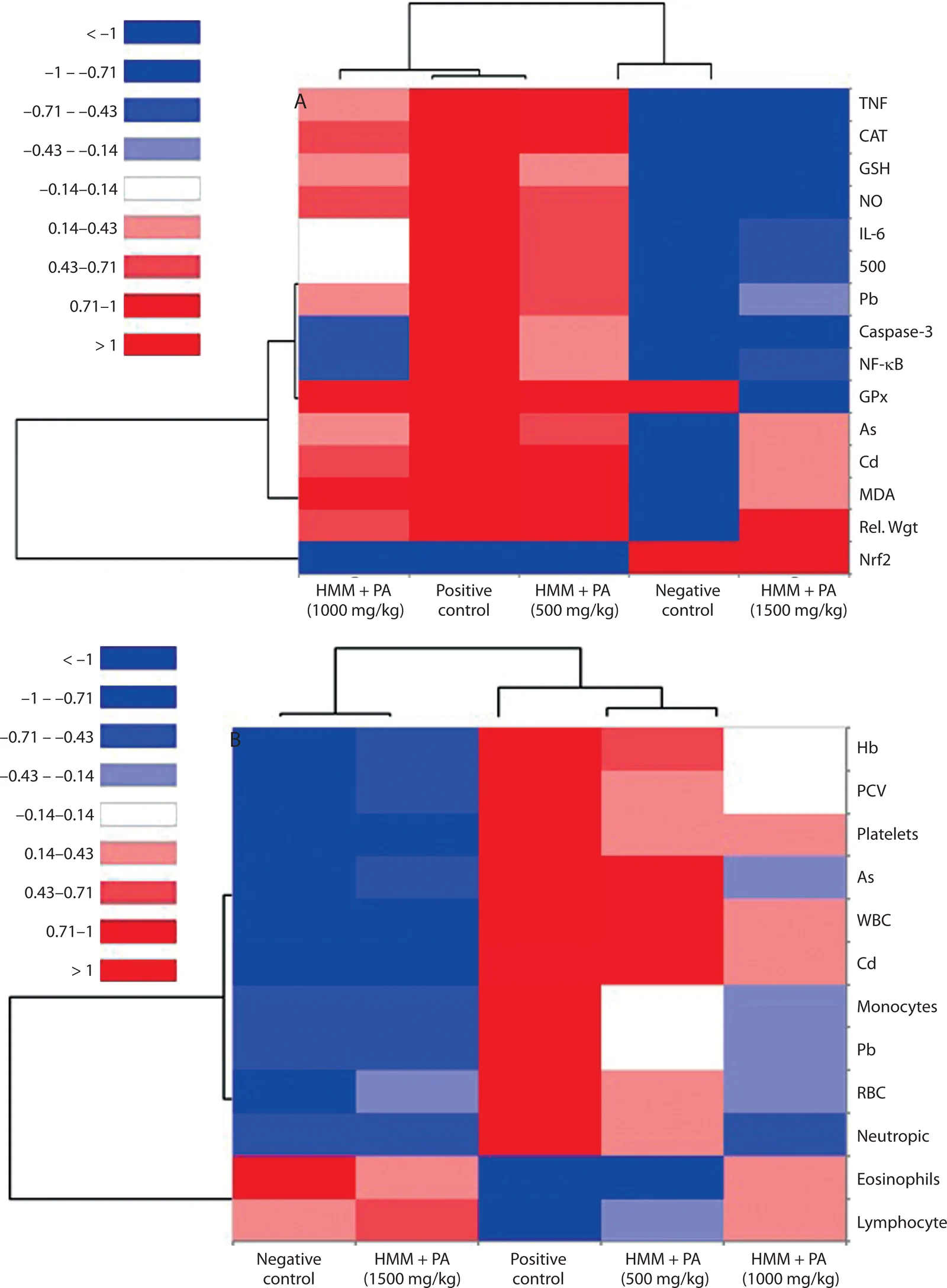
Hierarchical cluster analysis depicting the toxic and mitigating effects of HMM and PA treatment on biochemical parameters in animal lung (**A**) and blood (**B**). As – arsenic, CAT – catalase, Cd – cadmium, GPx – glutathione peroxidase, GSH – reduced glutathione, Hb – hemoglobin, HMM – heavy metal mixture, IL-6 – interleukin-6, MDA – malondialdehyde, NF-κB – nuclear factor kappa B, NO – nitric oxide, Nrf2 – nuclear factor erythroid 2-related factor 2, PA – *Prosopis africana*, Pb – lead, PCV – packed cell volume, RBC – red blood cell, Rel. Wgt – relative lung weight, SOD – superoxide dismutase, TNF – tumor necrosis factor, WBC – white blood cell

Blood HCA showed similar patterns (Figure 8B). The HMM group exhibited a highly toxic profile with an elevated metal burden and impaired hematological parameters (lower PCV, RBC, Hb; higher WBC, platelets, neutrophils). The 1000 and 1500 mg/kg PA doses clustered with the controls, indicating complete normalization of circulating parameters, while the 500 mg/kg dose remained partially associated with the HMM group.

These findings confirm that HMM induces a unified toxic state across the lung and blood compartments, characterized by oxidative-inflammatory stress and hematological disruption, which PA systematically reverses in a dose-dependent manner.

HCA was performed on *Z*-score-standardized data. Protective markers (Nrf2, PCV, RBC, SOD, CAT, GSH, GPx, Hb) were reverted (by multiplying by −1). Red signifies the worst overall state (high toxicity or low protection), and blue signifies the best overall state. The dendrograms show that the HMM-only control clusters distinctly, while the PA-treated groups shift progressively toward the negative control profile, confirming dose-dependent mitigation of HMM-induced systemic pathology in both tissues.

## Discussion

HMM exposure poses severe health risks in industrialized and mining regions, triggering bioaccumulation, oxidative stress, inflammation, and multiorgan damage (Hou et al. 2025). This study demonstrates that PA extract provides multilevel protection against HMM toxicity. It acts by reducing metal bioavailability – putatively via chelation and/or altered absorption – as well as antioxidant and anti-inflammatory mechanisms. This coordinated action restores biochemical, hematological, and histological homeostasis.

The dose-dependent reduction of tissue and blood metal burdens by PA, with the highest dose achieving > 85% reduction, suggests that upstream modulation of metal bioavailability is a primary protective mechanism. This process may involve chelation by polyphenolic constituents, reduced intestinal absorption, or enhanced excretion, although direct evidence from metal–ligand binding studies or urinary/fecal excretion measurements was not obtained. The stronger effect in blood versus lung tissue indicates that PA primarily intercepts circulating metals, preventing tissue deposition by limiting their bioavailability (Figure 2). The polyphenolic constituents of PA – rich in hydroxyl and carboxyl groups – can chelate divalent and trivalent metal ions (Singh and Sharma 2020), forming stable, excretable complexes that limit intestinal absorption and enhance mobilization of tissue-sequestered metals (Ceramella et al. 2024; Gupta et al. 2015). By limiting free metal ions, this upstream intervention mechanically blocks the downstream oxidative-inflammatory cascade. Comparable hepato-renal protection studies have confirmed that PA reduced metal burden and improved oxidative-inflammatory endpoints via Nrf2/NF-κB modulation (Ozoani et al. 2025). Additionally, a study on a neuroprotective rat model demonstrated that PA reduced heavy metal accumulation in neural tissues, while enhancing antioxidant enzyme activity (Orisakwe et al. 2024).

The observed oxidative stress – characterized by elevated lipid peroxidation, increased nitrosative stress, and depleted antioxidant defenses – reflects metal-catalyzed ROS generation and inactivation of sulfhydryl-dependent enzymes (Jomova et al. 2025). PA’s restoration of the oxidative-antioxidant balance at the highest (1500 mg/kg) dose suggests a dual action: direct ROS scavenging by polyphenolic constituents and the up-regulation of endogenous antioxidant enzyme expression, possibly via Nrf2 activation. This coordinated action suggests that the metal chelation activity of PA synergizes with its antioxidant activity, breaking the self-perpetuating cycle of metal-induced ROS generation and antioxidant depletion observed in neurotoxic and hepatorenal models (Orisakwe et al. 2024; Ozoani et al. 2025).

Despite a 39-fold compensatory elevation in Nrf2 protein expression under HMM stress – representing an emergency transcriptional response to oxidative overload – antioxidant enzyme activities remained profoundly suppressed (Figure 4). This indicates the functional impairment of Nrf2 nuclear translocation and/or ARE-binding activity, rather than effective pathway activation. The dissociation between protein overexpression and downstream enzyme suppression confirms that heavy metals disrupt Nrf2 nuclear import (Barber et al. 2023). PA treatment dose-dependently normalized Nrf2 protein toward baseline levels (95.2% normalization at 1500 mg/kg; Table 1). This is consistent with the reestablishment of homeostatic Nrf2 regulation as PA’s exogenous antioxidant activity relieved the oxidative burden. Because our ELISA-based Nrf2 measurements reflect total protein in tissue homogenates, they do not directly quantify nuclear translocation or ARE-binding. Future studies employing nuclear fractionation or chromatin immunoprecipitation assays would provide direct mechanistic confirmation. PA’s substantial, dose-dependent restoration of the Nrf2/NF-κB balance (approaching control values) and its suppression of caspase-3 activity suggest that transcriptional reprogramming is a central protective mechanism. This reciprocal modulation of pro-oxidant (NF-κB) and antioxidant (Nrf2) transcriptional programs represents a key molecular switch underlying PA’s cytoprotective effects, aligning with known crosstalk where oxidative stress activates NF-κB while suppressing Nrf2 nuclear translocation and ARE-binding (Ozoani et al. 2025).

Histological integrity is a critical endpoint for assessing therapeutic efficacy. In this study, exposed animals showed severe lung damage, characterized by perivascular congestion, intraluminal BCs, and disrupted alveolar structure (Figure 5). Conversely, the PA (1500 mg/kg) group exhibited near-normal pulmonary histoarchitecture, with resolved congestion and no BCs. The nonsignificant reduction in lung weights, despite histological improvement, suggests gross morphometric changes behind cellular recovery, or that the 60-day treatment period was too short to completely resolve tissue edema. Alternatively, the relatively small sample size (*n* = 5) may have limited statistical power to detect modest changes (Figure 3). Longer treatment durations or the evaluation of tissue remodeling markers (e.g., matrix metalloproteinases and collagen deposition) would provide deeper insight into the temporal sequence of structural restoration. These histological findings link molecular/biochemical restoration to visible tissue repair. Similar recovery of tissue architecture following natural product administration has been documented in other plant studies (Bolat et al. 2025; Ma et al. 2025).

The inflammatory-apoptotic perturbations extended systemically as hematological changes. Some of these changes – anemia, thrombocytosis, leukocytosis – reflect systemic metal toxicity via oxidative damage to erythrocyte membranes, disrupted erythropoiesis due to inhibited heme synthesis, and acute inflammation (Yuan et al. 2025). HMM caused shifts in leukocyte differentials: lymphocytes declined while neutrophils and monocytes rose. PA restored these parameters, showing protection beyond the primary target organ (lung) to systemic hematopoietic function.

Multivariate correlation analyses reinforced the associations between metal burden and downstream pathological cascades. Strong positive correlations between tissue metal levels and oxidative-inflammatory markers (*r* > 0.8), coupled with inverse associations with antioxidant defenses (*r* < –0.85), confirm that metal accumulation is the primary driver of toxicity. Similarly, blood metal levels correlated with hematological and immune dysregulation, supporting a unified systemic response to HMM exposure (Ajarem et al. 2022; Beglari et al. 2025).

Multivariate pattern recognition provided systemslevel validation of PA’s protective efficacy. PCA revealed that PC1, explaining > 83% of variance, clearly separated HMM-exposed animals (negative PC1 loadings for metals, oxidative stress, and inflammation) from PA-treated and control groups (positive PC1 loadings for antioxidant enzymes and hematological recovery). Hierarchical clustering confirmed this separation; HMM-exposed animals formed a discrete toxicity cluster, while PA-treated groups shifted dose-dependently toward the controls. Notably, the 1500 mg/kg group clustered closest to the controls in both the lung and blood datasets, providing unsupervised, systems-level evidence of near-complete physiological restoration (Ozoani et al. 2024).

The integrated mechanistic profile of PA – upstream metal chelation coupled with downstream Nrf2/NF-κB modulation – distinguishes it from conventional single-target chelators like EDTA, which lack antioxidant or anti-inflammatory activity (Fulgenzi et al. 2020). This multi-target approach may be effective in managing chronic, low-level mixed metal exposures where oxidative-inflammatory damage persists despite chelation. The dose-dependent efficacy of PA (optimal at 1500 mg/kg in rats) allows for allometric scaling to human equivalent doses, though pharmacokinetic validation remains necessary (Kumar et al. 2018; Singh and Sharma 2020). The doses 500–1500 mg/kg warrant explicit translational consideration. Applying standard allometric scaling (Reagan-Shaw et al. 2008), the 1500 mg/kg rat dose corresponds to a human dose of ~243 mg/kg (~17 g for a 70-kg adult), which is high for practical clinical use. This reinforces the need for bioassay-guided fractionation to concentrate bioactive constituents and reduce the required therapeutic dose, as well as to optimize formulation for improved bioavailability. In West African ethnomedicine, PA was typically used as aqueous decoctions at lower effective doses. This suggests that constituent enrichment and delivery optimization are key translational priorities.

These findings position PA as a potential phytotherapeutic adjunct for heavy metal toxicity management, particularly in resource-limited settings where synthetic chelators are inaccessible or expensive. The multi-target mechanism of PA supports its potential applications beyond acute poisoning, including chronic occupational exposures and environmental remediation. Future clinical translation should prioritize: 1) standardized formulation with quality control for active constituents; 2) phase I safety and pharmacokinetic studies in healthy volunteers; 3) bioequivalence and bioavailability optimization; 4) comparative efficacy trials against standard chelators in metal-exposed populations; and 5) development of combination therapies leveraging PA’s antioxidant capacity with conventional chelation. Although preliminary safety data based on the traditional use of PA in West African ethnomedicine are available, formal toxicity evaluations remain essential.

Several limitations warrant consideration. First, using a crude extract precludes the identification of specific bioactive constituents. Future bioassay-guided fractionation coupled with LC–MS/MS should isolate the primary chelating and Nrf2-activating compounds. Second, pharmacokinetic parameters remain undefined. Critically, the proposed chelation mechanism was not directly validated by collecting urinary or fecal metal excretion data or conducting metal–ligand binding assays. The observed reductions in tissue and blood metal concentrations may, therefore, reflect altered absorption, redistribution, or enhanced excretion rather than – or in addition to – direct chelation. Future studies should incorporate these measurements to rule out the above possibilities. Third, our 60-day subacute exposure model may not fully represent chronic environmental exposures or capture long-term safety concerns. Fourth, histopathological assessment was purely descriptive. Semiquantitative scoring systems (e.g., graded indices for vascular congestion, alveolar inflammation, and structural disruption) and morphometric analyses were not applied. Future studies should incorporate predefined, blinded scoring rubrics to quantitatively support structural observations. Fifth, the use of only male rats to minimize hormonal variability is a significant limitation, as sex-specific differences in heavy metal toxicokinetics (e.g., higher Cd retention in females due to lower ferritin stores), Nrf2 signaling, and baseline antioxidant enzyme activity are well-documented. Thus, the protective effects of PA may not directly extrapolate to female subjects, and the conclusions should be interpreted within this constraint. Future studies should include both sexes and examine potential hormonal interactions with PA’s protective mechanisms. Sixth, the crude methanolic extract was not subjected to batch-specific quantitative phytochemical characterization (e.g., total phenolic or flavonoid content), which limits reproducibility and precludes attribution of observed effects to specific constituent classes. Bioassay-guided fractionation coupled with LC–MS/MS profiling is recommended in future work. Seventh, while our dose range (500–1500 mg/kg) showed efficacy, formal dose-optimization and toxicity ceiling studies are needed. Eighth, the sample size (*n* = 5 per group) – though aligned with 3Rs-guided principles and regulatory norms for sub-chronic rodent toxicity studies (OECD TG 407) – limits statistical power, particularly for multivariate analyses like PCA and hierarchical clustering, which are more robust with larger datasets. The multivariate findings presented here are therefore exploratory and hypothesisgenerating rather than confirmatory, and replication in adequately powered studies is recommended. Finally, human translation requires dose-extrapolation studies, clinical pharmacology evaluation, and regulatory toxicology assessments, especially since traditional PA use lacks standardization.

## Conclusions

PA extract confers multi-level protection against HMM toxicity by reducing metal burden – likely via chelation and/or reduced bioavailability – and driving transcriptional reprogramming. By lowering metal bioaccumulation and modulating the Nrf2/NF-κB axis, PA systematically reverses HMM-induced oxidative stress, inflammatory signaling, apoptotic activation, and hematological disruption. The dose-dependent restoration of lung histoarchitecture, backed by integration of biochemical, hematological, and multivariate pattern analyses, confirms the therapeutic efficacy of PA.

These findings, obtained in male rats, position PA as a mechanistically distinct alternative to conventional chelators, offering cytoprotection beyond metal sequestration, though generalizability to female subjects and other populations requires further investigation. Clinical translation requires identifying bioactive constituents, defining pharmacokinetics, and validating chronic safety. However, PA’s traditional use, preliminary safety profile, and multi-target mechanism make it a compelling candidate for managing heavy metal toxicity in highexposure populations. Future work should prioritize standardization, human dose extrapolation, and comparative efficacy trials against existing therapies to promote the clinical use of PA.
